# Hepatocellular Carcinoma Patients With Performance Status 1 Deserve New Classification and Treatment Algorithm in the BCLC System: Erratum

**DOI:** 10.1097/01.md.0000471206.87982.00

**Published:** 2015-08-21

In the article “Hepatocellular Carcinoma Patients With Performance Status 1 Deserve New Classification and Treatment Algorithm in the BCLC System”,^1^ which appeared in Volume 94, Issue 29 of *Medicine*, the affiliations footnote on page 1 was presented incorrectly. The article has since been corrected online.
